# Knowledge, attitudes, and practices related to traditional and conventional ectoparasite control in domestic ruminants: a cross-sectional survey conducted in Hawassa City, Ethiopia

**DOI:** 10.1186/s12917-026-05572-y

**Published:** 2026-05-23

**Authors:** Kabech Gedeno Gemechu, Wosenyelesh Kebede Ali, Mihiret Genene Zewde

**Affiliations:** 1 Faculty of Veterinary Medicine, College of Natural and Computational Sciences, Hawassa University, P.O. Box 05, Hawassa, Ethiopia; 2 Department of Statistics, College of Natural and Computational Sciences, Hawassa University, P.O. Box 05, Hawassa, Ethiopia

**Keywords:** Attitude, Conventional medicine, Knowledge, Practice, Ruminant ectoparasites, Traditional medicine

## Abstract

**Background:**

Ruminant livestock is an integral component of agricultural production in Ethiopia. However, productivity is constrained by ectoparasites such as ticks, lice, fleas, and mites, which cause economic losses and transmit pathogens. In this study, traditional methods refer to ethnoveterinary remedies based on indigenous knowledge, whereas conventional methods refer to commercially manufactured ectoparasiticides.

**Objective:**

This study aimed to assess community knowledge, attitudes, and practices (KAP) regarding traditional and conventional ectoparasite control methods in domestic ruminants, identify associated risk factors, and document treatments known in Hawassa City, Ethiopia.

**Methods:**

A cross-sectional questionnaire-based survey was conducted from January to June 2025. Data were collected through face-to-face interviews using Kobo Toolbox from 423 randomly selected participants. Data were analyzed using STATA version 17, and binary logistic regression was used to identify factors associated with KAP scores.

**Results:**

Of the 423 participants, 167 (40.0%, 95% CI: 34.8–44.3) were aware of both traditional and conventional treatments, 107 (25.0%, 95% CI: 21.2–29.7) knew only traditional treatments, 48 (11.0%, 95% CI: 8.5–14.8) knew only conventional treatments, and 101 (24.0%, 95% CI: 19.9–28.2) had no knowledge of any treatments. Regarding traditional treatments, 35.5%, 38.1%, and 40.4% of respondents demonstrated good knowledge, positive attitudes, and good practices, respectively. For conventional treatments, the corresponding proportions were 73.8%, 21.0%, and 53.4%, respectively. Logistic regression analysis indicated that age, education level, occupation, religion, farming system, and farming experience were significantly associated with KAP scores for traditional treatments (*P* < 0.05). For conventional treatments, age, sex, religion, marital status, and farming experience were significant predictors. Additionally, the study identified ten reasons for the use of traditional treatments, fourteen types of traditional remedies, and seven types of conventional treatments.

**Conclusion:**

Knowledge, attitudes, and practices regarding traditional ectoparasite control methods were generally low, whereas knowledge and practices related to conventional methods were higher, although attitudes remained low. These findings highlight the need for targeted community awareness programs to promote the safe and informed use of both traditional and conventional ectoparasite control methods.

**Supplementary Information:**

The online version contains supplementary material available at 10.1186/s12917-026-05572-y.

## Introduction

The livestock sector is a key component of global agriculture, contributing approximately 40% of agricultural output in developed countries and 20% in developing countries, while supporting the livelihoods and food security of billions of people worldwide [1–3]. Ethiopia possesses the largest livestock population in Africa, with an estimated 66.3 million cattle, 38.1 million sheep, 45.7 million goats, 41.4 million poultry, 12.6 million equines, 7.0 million camels, and 6.0 million beehives [4]. Reflecting this substantial resource base, the country produces approximately 4.96 billion liters of cow milk, 2.43 billion liters of camel milk, 268.8 million eggs, and 129.3 million kilograms of honey annually [5].

Ruminant livestock constitute the largest proportion of Ethiopia’s national herd and provide essential socioeconomic and ecological services. They contribute to poverty alleviation, supply meat and milk, generate income from hides and skins, and serve as financial assets during seasonal needs and emergencies [6–8]. In addition, they support ecosystem management, improve soil fertility through manure, provide draft power, and enhance household wealth and social status [1, 7, 9]. Small ruminants are particularly important for food security and household income, contributing significantly to nutrition and rural livelihoods [1, 7, 8]. Furthermore, ruminant production contributes to national export earnings through live animal and meat exports [1, 6, 8].

Despite their importance, ruminant production in Ethiopia is constrained by multiple factors, particularly parasitic diseases. Ectoparasites, including ticks, lice, fleas, and mites, are widely distributed in cattle, sheep, and goats across the country [1, 6, 9–11]. These parasites cause substantial economic losses through increased morbidity and mortality, reduced productivity, and the downgrading or rejection of hides and skins [12–16]. They also reduce outputs of meat, milk, manure, and draft power, thereby negatively affecting overall livestock productivity [9, 10, 12, 14–16].

In addition to economic impacts, ectoparasites significantly compromise animal health and welfare by causing irritation, inflammation, anemia, tissue damage, and, in severe cases, death [10–12, 14, 16]. By feeding on blood and damaging the skin, they create wounds and persistent discomfort, reducing productivity and lowering the quality of hides and skins. Ectoparasite infestation is a major cause of skin lesions in small ruminants, resulting in substantial losses to farmers and the tanning industry [11, 12, 14, 15]. Moreover, ectoparasites act as vectors of protozoan, bacterial, and rickettsial pathogens of veterinary and zoonotic importance [17–20].

To control ectoparasite infestations, livestock owners in Ethiopia use both traditional and conventional approaches [21–24]. Traditional approaches refer to ethnoveterinary practices based on indigenous knowledge and locally available materials, such as medicinal plants, ash, butter, kerosene, smoke, and manual tick removal. These methods are widely used due to their affordability, accessibility, and cultural acceptance, particularly in rural areas [23–25]. In contrast, conventional approaches involve commercially manufactured veterinary products, including acaricides and insecticides applied as dips, sprays, injections, or pour-on formulations. Their effectiveness depends on correct application, and their use may be constrained by cost, availability, and emerging drug resistance [22, 26–28].

Despite the availability of these control methods, ectoparasite management in Ethiopia faces several challenges, including reduced treatment efficacy, safety concerns, drug resistance, and risks to human health and the environment [21, 22, 26–30]. Consequently, ectoparasite infestations remain highly prevalent in many regions, including Hawassa City [10, 22, 29, 30]. Contributing factors include inappropriate use of treatments, limited knowledge of ectoparasite biology, incorrect drug selection, misconceptions about efficacy, and poor management practices such as underdosing, overdosing, and irregular application [21, 22, 24, 26, 28, 30, 31].

Addressing these challenges requires targeted interventions informed by community knowledge, attitudes, and practices (KAP). However, KAP data on ectoparasite control remain limited in Ethiopia. Apart from a study conducted in South Omo Zone focusing on acaricide use and tick control [22], comprehensive assessments are scarce. Therefore, this study was conducted in Hawassa City to generate baseline information on community knowledge, attitudes, and practices regarding traditional and conventional ectoparasite control methods in domestic ruminants.

Hawassa City was selected as it represents an important urban and peri-urban livestock production setting in southern Ethiopia, where traditional ethnoveterinary practices coexist with modern veterinary services. Livestock owners in the area have access to both indigenous knowledge and commercially available veterinary products, making it suitable for comparing traditional and conventional ectoparasite control practices. In addition, the area supports diverse production systems and substantial populations of cattle, sheep, and goats, and previous studies have documented ectoparasite presence in the region [10, 11, 29, 30].

## Materials and methods

### Description of the study area

The study was conducted from January to June 2025 to assess community knowledge, attitudes, and practices (KAP) regarding traditional and conventional medicines for the control of ectoparasites in domestic ruminants in Hawassa City. Hawassa City, the capital of the Sidama Region in southern Ethiopia, is located approximately 273 km south of Addis Ababa at 7°03′ N latitude and 38°28′ E longitude, with an elevation of about 1,708 m above sea level [32].

The city has a sub-humid temperate climate, with mean monthly temperatures ranging from 17 to 20 °C and an average annual rainfall of approximately 1,128 mm. Recent studies have reported increasing temperature variability in southern Ethiopia, which may influence ecological dynamics and livestock health [32, 33]. Hawassa City was selected due to its importance as an urban livestock production area and previously reported ectoparasite burden [29, 30].

According to official reports, the Sidama Region has an estimated ruminant population of 2,492,158 cattle, 470,338 sheep, and 486,262 goats [4]. Within Hawassa City, the livestock population is estimated at 165,306 cattle, 58,176 sheep, and 66,112 goats. The total human population is estimated at 525,053, of which 363,886 reside in urban areas and 161,167 in rural areas [34].

**Fig. 1:**
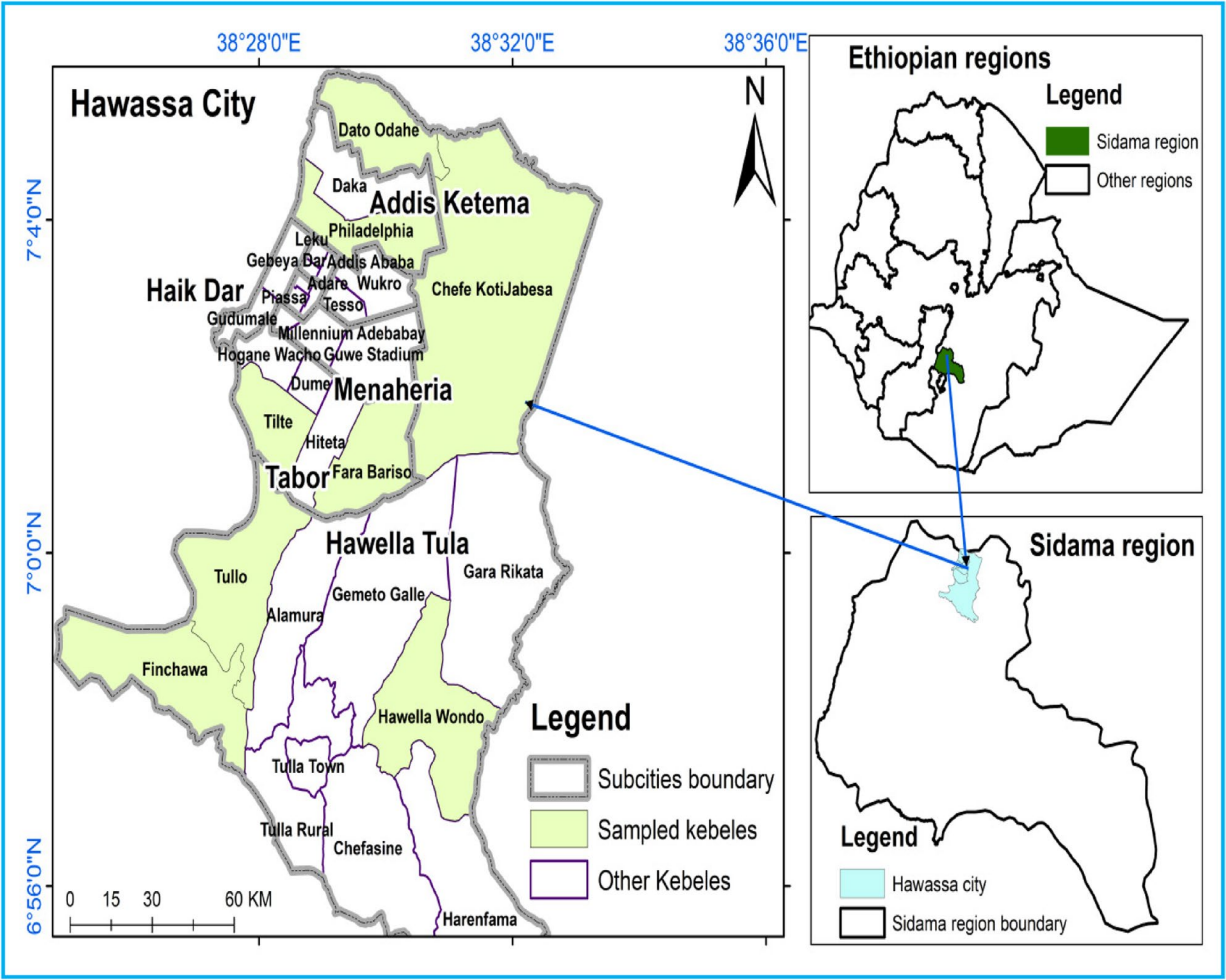
Map of Hawassa City, Sidama Region, Ethiopia, showing the selected sub-cities and kebeles included in the study

Hawassa City covers approximately 50 km² and is administratively divided into eight sub-cities and 32 kebeles. A kebele is the smallest administrative unit in Ethiopia and serves as the primary level of local governance and service delivery. The study was conducted among 423 ruminant-owning households selected from eight randomly chosen kebeles (Fig. 1).

## Study subjects

The study population comprised households engaged in ruminant production (cattle, sheep, and goats) under different management systems. Eligible respondents were household members aged 18 years or older responsible for ruminant management and willing to participate. Only households owning at least one ruminant were included.

### Study design

A community-based cross-sectional questionnaire survey was employed.

### Sample size determination

The required sample size was determined using the formula for estimation of a single population proportion described by Michael Thrusfield [35]. Since no previous study had investigated knowledge, attitudes, and practices related to traditional and conventional ectoparasite control in the study area, an expected prevalence (Pexp) of 50% was assumed to obtain the maximum sample size. The calculation was performed using a 95% confidence level (Z = 1.96) and a desired absolute precision of 5% (d = 0.05):

$$\:n=\frac{{\mathrm{Z}}^{2}\times\:\mathrm{P}\mathrm{e}\mathrm{x}\mathrm{p}\times\:\:(1-\mathrm{P}\mathrm{e}\mathrm{x}\mathrm{p})}{{d}^{2}}$$

Where:

n = required sample size.

Z = standard normal deviate corresponding to the desired confidence level.

Pexp = expected prevalence.

d = desired absolute precision.

Substituting the values:

$$n = \frac{1.96^2 \times 0.50 \times (1 - 0.50)}{(0.05)^2} = 384$$

To account for possible non-response and incomplete questionnaires, a 10% contingency was added:

$$\text{Final sample size } = 384 + (384 \times 0.10) = 422.4 \approx 423$$

Accordingly, the final target sample size was 423 respondents.

### Sampling procedure

A multistage sampling technique was employed to select study participants. First, three sub-cities of Hawassa were purposively selected based on their relatively greater dependence on livestock production. Subsequently, eight kebeles were randomly selected from these sub-cities. All ruminant-owning households within the selected kebeles (*n* = 7,321) constituted the primary sampling frame. Households that met the inclusion criteria and consented to participate were eligible for the study.

The final sample size of 423 households was proportionally allocated to each kebele according to the number of ruminant-owning households. The sample size for each kebele was calculated using:

$$\mathrm{ni} = \frac{Ni}{N} \times n$$

Where:

ni = allocated sample size for kebele i.

Ni = total number of ruminant-owning households in kebele i.

N = total number of ruminant-owning households in all selected kebeles (7,321).

n = total sample size (423).

Systematic random sampling was then used to select households within each kebele. The sampling interval (k) for each kebele was calculated as:

$$\mathrm{k} = \frac{Ni}{\mathrm{ni}}$$

The first household was selected by simple random sampling using a lottery method, and every kth household thereafter was included until the required sample size for each kebele was reached.

### Data collection tool and procedure

The questionnaire used in this study was specifically developed for the present research and has not been previously published. It was designed following an extensive review of published literature on ectoparasite control practices and Knowledge, Attitudes, and Practices (KAP) studies in veterinary public health, along with the researchers’ field experience. The tool aimed to capture information on both traditional and conventional ectoparasite control methods among domestic ruminant owners.

The questionnaire was initially developed in English and then translated into the local language. To ensure linguistic accuracy and conceptual equivalence, the translated version was reviewed by bilingual experts familiar with veterinary terminology. The instrument was pre-tested on eight ruminant-owning households outside the study area to assess clarity, consistency, and cultural appropriateness. Based on the pre-test results, minor revisions were made to improve wording and the logical flow of questions without altering the overall structure or content. The final questionnaire was easily understood by respondents and effectively captured the intended information. The English version of the questionnaire is provided in Additional file 1.

The final questionnaire comprised three sections. Section I addressed socio-demographic characteristics and ruminant production systems (26 items), including herd size, farming system, shelter management, and access to veterinary services. Section II assessed knowledge (13 items), attitudes (14 items), and practices (7 items) related to traditional ectoparasite control methods, along with reasons for their use. Section III assessed knowledge (8 items), attitudes (7 items), and practices (6 items) regarding conventional ectoparasite control methods.

The questionnaire included multiple-choice, open-ended, closed-ended, dichotomous (yes/no), and five-point Likert-scale items. Likert-scale responses ranged from “strongly disagree” to “strongly agree” for knowledge and attitude statements, and from “always” to “never” for practice statements.

Prior to data collection, meetings were held with kebele administrators, livestock development agents, and local agricultural offices to explain the study objectives. Eligible households were identified with the support of local animal health professionals to ensure inclusion of active ruminant owners.

Data were collected through face-to-face interviews conducted at respondents’ households in the local language. Before each interview, participants were informed about the study objectives, confidentiality of responses, voluntary participation, and their right to withdraw at any time without consequences. Verbal informed consent was obtained prior to participation. Data were recorded electronically using Kobo Collect to enhance data accuracy, minimize entry errors, and ensure secure data management.

### Data management and analysis

All questionnaires were completed successfully, resulting in a 100% response rate. Data were downloaded from Kobo Collect into Microsoft Excel, cleaned, coded, and then exported to STATA version 17 for analysis. Descriptive statistics were computed for all variables and summarized using frequencies and percentages. Blank and “don’t know” responses were excluded from the analysis.

KAP (Knowledge, Attitude, and Practice) scores for traditional and conventional medicines were calculated by summing responses within each domain and dividing by the total number of questions in that domain to obtain mean scores. Responses were measured on a five-point Likert scale ranging from 1 to 5. Mean scores below 2.5 (< 50%) were classified as poor KAP, while mean scores ≥ 2.5 (≥ 50%) were classified as good KAP.

Six dependent variables were defined: knowledge, attitude, and practice related to traditional medicines, and knowledge, attitude, and practice related to conventional medicines. Thirteen demographic variables were considered as independent variables. Univariable binary logistic regression was initially used to screen associations between explanatory variables and outcome variables. Variables with *P* < 0.05 in the univariable analysis were included in multivariable binary logistic regression models. A backward stepwise elimination approach was applied, and associations were considered statistically significant at *P* < 0.05 with 95% confidence intervals.

## Results

### Demographic characteristics of respondents

A total of 423 ruminant-owning households participated in the study. Of these, 239 (56.5%, 95% CI: 51.6–61.3) were male and 184 (43.5%, 95% CI: 38.7–48.4) were female. The largest proportion of respondents were aged 36–60 years (46.3%), followed by 20–35 years (40.0%) and > 60 years (13.7%). Most respondents were married (73.8%), while 18.9% were single and 7.3% were widowed. Regarding education, 41.6% were illiterate, 30.0% had primary education, 17.3% secondary, and 11.1% tertiary education. The majority were Protestant (63.4%), and farming was the main occupation for 52.2% of respondents. Most participants had 1–10 years of farming experience (44.0%), and the intensive production system was the most common (42.3%) (Table 1).

**Table 1:** Socio-demographic characteristics of respondents in Hawassa City, Ethiopia (*n* = 423)

Variable	Category	Frequency	Percentage (%)	95% CI (%)
Sex	Male	239	56.5	51.6–61.3
	Female	184	43.5	38.7–48.4
Age	20–35	169	40.0	35.3–44.8
	36–60	196	46.3	41.5–51.2
	> 60 years	58	13.7	10.6–17.4
Marital status	Married	312	73.8	69.3–77.9
	Single	80	18.9	15.3–23.0
	Widowed	31	7.3	5.0–10.2
Education	Illiterate	176	41.6	36.9–46.5
	Primary	127	30.0	25.7–34.6
	Secondary	73	17.3	13.8–21.2
	Tertiary	47	11.1	8.3–14.5
Religion	Protestant	268	63.4	58.6–68.0
	Orthodox	57	13.5	10.4–17.1
	Muslim	40	9.4	6.8–12.7
	Catholic	58	13.7	10.6–17.4
Occupation	Farmer	221	52.2	47.4–57.1
	Employee	92	21.8	17.9–26.0
	Merchant	110	26.0	21.9–30.5
Farming experience	1–10	186	44.0	39.2–48.8
	11–30	176	41.6	36.9–46.5
	> 30 years	61	14.4	11.2–18.1
Farming system	Intensive	179	42.3	37.6–47.2
	Semi-intensive	83	19.6	15.9–23.7
	Extensive	161	38.1	33.4–42.9
Total		**423**	**100.0**	

*Employee* Government employees

### Household ruminant livestock ownership patterns and herd sizes

Most households in Hawassa City kept either cattle only (37.6%) or cattle and goats (35.0%), while fewer households maintained sheep only (2.4%), goat only (5.7%), cattle and sheep (3.8%), sheep and goats (3.1%), or all three species (12.5%). The median herd size varied across ownership groups, ranging from 4 animals in sheep-only and goat-only households to 12 animals in households keeping all three species, indicating substantial variation in herd composition and size. In some cases, the median herd size did not correspond to an observed individual household value due to the distribution of data within groups (Table 2).

**Table 2:** Household ruminant livestock ownership patterns and herd sizes in Hawassa City (*n* = 423)

RLG	*n* (%) [95% CI]	TNA	MNA	HM	Min	Max
Cattle only	159 (37.6%) [33.0–42.4]	1,169	5	28	1	45
Sheep only	10 (2.4%) [1.1–4.3]	54	4	0*	1	16
Goat only	24 (5.7%) [3.7–8.3]	108	4	0*	1	15
Cattle & Sheep	16 (3.8%) [2.2–6.1]	169	7.5	1–2**	2	32
Cattle & Goat	148 (35.0%) [30.4–39.7]	1,562	8	22	1	58
Sheep & Goat	13 (3.1%) [1.6–5.2]	90	5	1	2	18
Cattle, Sheep & Goat	53 (12.5%) [9.5–16.1]	659	12	7	2	36
Total	**423 (100%)**	**3**,**811**	**6**	–	**1**	**58**

The median represents the central value of ordered observations and may not correspond to an observed household value

*RLG* Ruminant Livestock Group, *n* Number of respondents, *%* Percentage, *95% CI* 95% confidence interval, *TNA* Total number of animals, *MNA* Median number of animals, *HM* Number of households with values at or around the median, *Min* Minimum number of animals, *Max* Maximum number of animals

0* = No household had exactly the median herd size, but the median still represents the middle value of all households

1–2** = The median falls between two households’ herd sizes, so 1–2 households have the values closest to the median (e.g., 7.5 for Cattle & Sheep)

### Knowledge of ruminant ectoparasite treatments

**Fig. 2:**
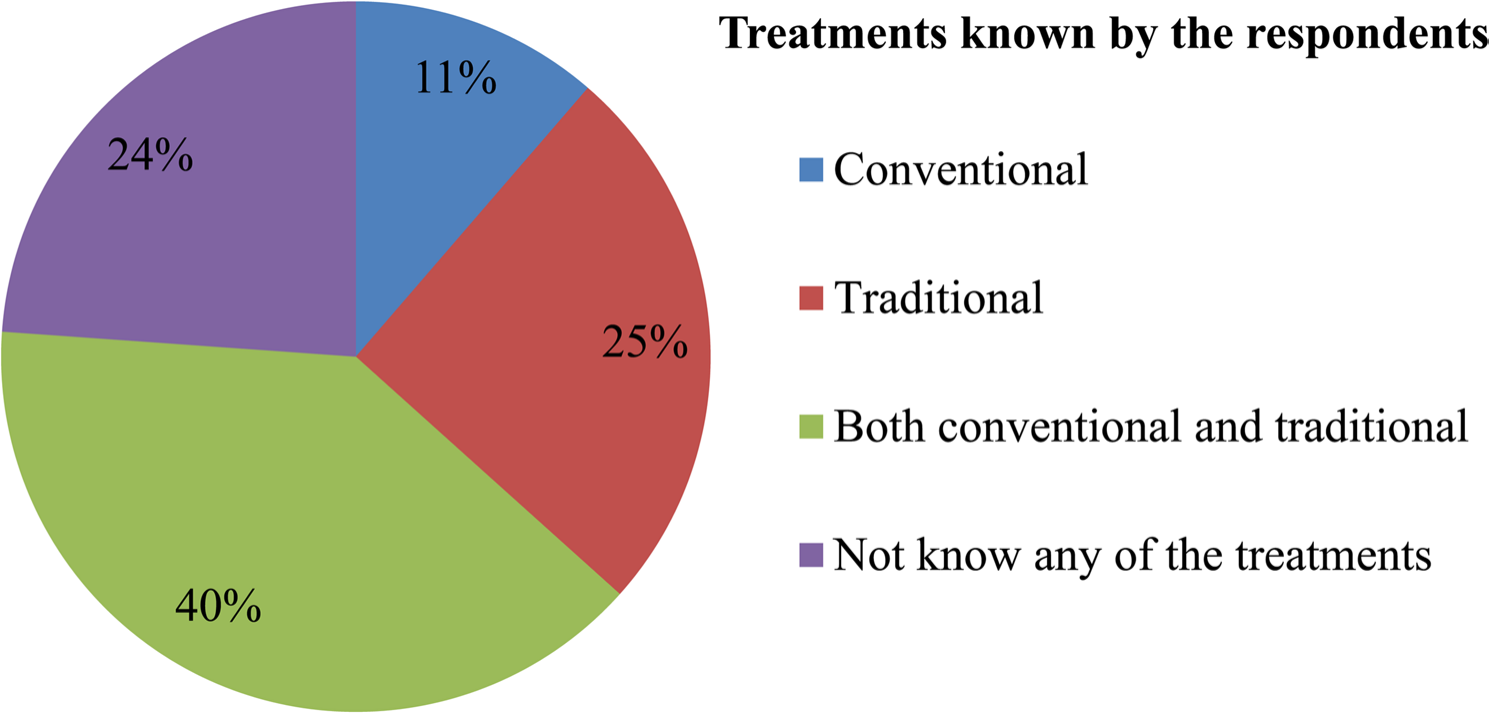
Knowledge of traditional and conventional ectoparasite treatments among respondents in Hawassa City (*n* = 423)

Among the 423 respondents, 167 (40.0%, 95% CI: 34.8–44.3) had knowledge of both traditional and conventional ectoparasite treatments. Additionally, 107 (25.0%, 95% CI: 21.2–29.7) reported knowledge only of traditional treatments, while 48 (11.0%, 95% CI: 8.5–14.8) knew only conventional treatments. A total of 101 respondents (24.0%, 95% CI: 19.9–28.2) reported no knowledge of any ectoparasite treatments (Fig. 2).

### Knowledge, attitudes, and practices regarding traditional medicines

#### Factors associated with knowledge

Of the total respondents, 150 (35.5%, 95% CI: 30.9–40.2) demonstrated good knowledge of traditional medicines, whereas 273 (64.5%, 95% CI: 59.8–69.1) had poor knowledge. Age, education, and farming experience were significantly associated with knowledge levels. Respondents aged 36–60 years and those older than 60 years were more likely to have good knowledge compared with those aged 20–35 years (OR = 1.98, 95% CI: 1.20–3.20, *P* = 0.006; OR = 5.58, 95% CI: 2.80–11.30, *P* < 0.001, respectively). Similarly, respondents with 11–30 years of farming experience were more likely to be knowledgeable than those with 1–10 years of experience (OR = 2.50, 95% CI: 1.50–4.10, *P* < 0.001). In contrast, respondents with primary education were less likely to have good knowledge compared with illiterate respondents (OR = 0.41, 95% CI: 0.20–0.70, *P* = 0.002) (Table 3).

**Table 3:** Factors significantly associated with KAP of traditional medicines (multivariable binary logistic regression, *n* = 423)

Var	Category	F	Knowledge				Attitude				Practice			
			G	*P*	OR(95% CI)	*P*-value	+Ve	_Ve	OR(95%CI)	*P*-value	G	*P*	OR(95%CI)	*P*-value
Age	20 − 35	169	37	132	Ref		47	122	Ref		51	118	Ref	
	36 − 60	196	74	122	1.98(1.2–3.2)	0.006	76	120	1.52(0.9–2.5)	0.099	83	113	1.64(1.0-2.6)	0.035
	> 60 years	58	39	19	5.58(2.8–11.3)	0.000	38	20	2.87(1.4–5.8)	0.004	37	21	4.21(2.1–8.3)	0.000
E	Illiterate	176	77	99	Ref		87	89	Ref		80	96	Ref	
	Primary	127	28	99	0.41(0.2–0.7)	0.002	33	94	0.34(0.2–0.6)	0.000	35	92	0.54(0.3–0.9)	0.022
	Secondary	73	28	45	1.04(0.6–1.9)	0.907	29	44	0.67(0.4–1.3)	0.213	36	37	1.41(0.8–2.6)	0.262
	Tertiary	47	17	30	1.12(0.6–2.3)	0.752	12	35	0.42(0.2-1.0)	0.038	20	27	1.41(0.7–2.9)	0.346
FE	1–10	186	43	143	Ref		36	150	Ref		61	125		
	11–30	176	77	99	2.50(1.5–4.1)	0.000	89	87	3.63(2.2–6.1)	0.000	79	97		
	> 30 years	61	30	31	1.91(1.0-3.7)	0.059	36	25	3.78(1.9–7.5)	0.000	31	30		
R	Protestant	268	86	182			90	178	Ref		95	173	Ref	
	Orthodox	57	25	32			24	33	1.61(0.8–3.2)	0.174	26	31	1.38(0.7–2.6)	0.307
	Muslim	40	15	25			19	21	2.33(1.1–5.1)	0.032	17	23	1.48(0.7-3.0)	0.280
	Catholic	58	24	34			28	30	1.98(1.0-3.8)	0.039	33	25	2.65(1.4–4.9)	0.002
FS	Intensive	179	51	128			48	131	Ref		63	116		
	SI	83	36	47			41	42	2.17(1.2-4.0)	0.014	40	43		
	Extensive	161	63	98			72	89	1.55(0.9–2.6)	0.101	68	93		
O	Farmer	221	88	133			96	125			102	119	Ref	
	Employee	92	27	65			32	60			29	63	0.53(0.3–0.9)	0.025
	Merchant	110	35	75			33	77			40	70	0.83(0.5–1.4)	0.463
Total		**423**	**150**	**273**			**161**	**262**			**171**	**252**		

*Var* Variable,*F* Frequency, *OR* Odds ratio, *CI* Confidence interval, *+Ve* Positive, *−Ve* Negative, *G* Good, *P* Poor, *FS* Farming system, *Ref* Reference category, *E* Education, *SI* Semi-intensive, *R *Religion, *O* Occupation, *FE* Farming experience, *Employee* Government employees

### Factors associated with attitudes

Overall, 161 respondents (38.1%, 95% CI: 33.4–42.9) demonstrated a positive attitude toward traditional medicines, while 262 (61.9%, 95% CI: 57.1–66.6) exhibited a negative attitude. Age, farming experience, religion, education, and farming system were significantly associated with attitude. Respondents older than 60 years were more likely to have a positive attitude compared with those aged 20–35 years (OR = 2.87, 95% CI: 1.40–5.80, *P* = 0.004). Farming experience of 11–30 years and > 30 years was strongly associated with positive attitudes (OR = 3.63, 95% CI: 2.20–6.10, *P* < 0.001; OR = 3.78, 95% CI: 1.90–7.50, *P* < 0.001, respectively). Muslim and Catholic respondents were more likely to have positive attitudes compared with Protestant respondents. Farmers practicing semi-intensive systems were also more likely to have positive attitudes than those practicing intensive systems (OR = 2.17, 95% CI: 1.20–4.00, *P* = 0.014). Conversely, respondents with primary and tertiary education were less likely to demonstrate positive attitudes (Table 3).

### Factors associated with practices

Good practices toward traditional medicines were observed in 171 respondents (40.4%, 95% CI: 35.7–45.3), while 252 (59.6%, 95% CI: 54.7–64.3) demonstrated poor practices. Age, education, religion, and occupation were significantly associated with practice levels. Respondents aged 36–60 years and those older than 60 years were more likely to exhibit good practices compared with younger respondents (OR = 1.64, 95% CI: 1.00–2.60, *P* = 0.035; OR = 4.21, 95% CI: 2.10–8.30, *P* < 0.001, respectively). Respondents with primary education were less likely to demonstrate good practices compared with illiterate respondents (OR = 0.54, 95% CI: 0.30–0.90, *P* = 0.022). Catholic respondents were more likely to report good practices than Protestant respondents (OR = 2.65, 95% CI: 1.40–4.90, *P* = 0.002). In contrast, government-employed respondents were less likely to demonstrate good practices compared with farmers (OR = 0.53, 95% CI: 0.30–0.90, *P* = 0.025) (Table 3).

### Knowledge, attitudes, and practices regarding conventional medicines

#### Factors associated with knowledge

Out of the 423 respondents, 312 (73.8%, 95% CI: 69.3–77.9) demonstrated good knowledge of conventional medicines, while 111 (26.2%, 95% CI: 22.1–30.7) had poor knowledge. Age, farming experience, and religion were significantly associated with knowledge levels. Respondents older than 60 years were more likely to have good knowledge compared with those aged 20–35 years (OR = 2.89, 95% CI: 1.10–7.50, *P* = 0.027). Similarly, respondents with more than 30 years of farming experience were more likely to be knowledgeable than those with 1–10 years of experience (OR = 2.60, 95% CI: 1.10–6.00, *P* = 0.025). In contrast, Orthodox respondents were less likely to have good knowledge compared with Protestant respondents (OR = 0.43, 95% CI: 0.20–0.80, *P* = 0.010) (Table 4).

**Table 4:** Factors significantly associated with KAP of conventional medicines: multivariable binary logistic regression (*n* = 423)

Var	Category	F	Knowledge				Attitude				Practice			
			G	*P*	OR(95%CI)	*P*-value	+Ve	_Ve	OR(95%CI)	*P*-value	G	*P*	OR(95%CI)	*P*-value
Age	20–35	169	121	48	Ref		26	143	Ref		90	79		
	36–60	196	139	57	0.93(0.6–1.5)	0.751	40	156	1.64(0.9-3.0)	0.098	100	96		
	> 60yrs	58	52	6	2.89(1.1–7.5)	0.027	23	35	3.96(1.9–8.2)	0.000	36	22		
R	Protestant	268	205	63	Ref		63	205	Ref		138	130	Ref	
	Orthodox	57	35	22	0.43(0.2–0.8)	0.010	11	46	0.70(0.3–1.5)	0.344	27	30	0.93(0.5–1.7)	0.814
	Muslim	40	30	10	1.02(0.5–2.2)	0.968	11	29	1.55(0.7–3.4)	0.271	20	20	1.07(0.5–2.1)	0.851
	Catholic	58	42	16	0.85(0.4–1.7)	0.636	4	54	0.23(0.1–0.7)	0.007	41	17	2.16(1.2-4.0)	0.015
M	Married	312	234	78			54	258	Ref		155	157	Ref	
	Single	80	52	28			23	57	2.65(1.4-5.0)	0.002	54	26	1.97(1.2–3.3)	0.012
	Widowed	31	26	5			12	19	2.39(1.0-5.5)	0.041	17	14	1.03(0.5–2.2)	0.942
FE	1–10	186	127	59	Ref		31	155			99	87		
	11–30	176	132	44	1.20(0.8–1.9)	0.448	44	132			104	72		
	> 30years	61	53	8	2.60(1.1-6.0)	0.025	14	47			23	38		
Sex	Male	239	176	63			47	192			115	124	Ref	
	Female	184	136	48			42	142			111	73	1.57(1.0-2.4)	0.032
Total		**423**	**312**	**111**			**89**	**334**			**226**	**197**		

*Var* Variable, *F* Frequency, *OR* Odds ratio, *CI* Confidence interval, *+Ve* ZPositive, *−Ve* Negative, *G *Good, *P* Poor, *FS* Farming system, *Ref* Reference category, *R* Religion, *FE* Farming experience, *M* Marital status

### Factors associated with attitudes

Only 89 respondents (21.0%, 95% CI: 17.3–25.2) demonstrated a positive attitude toward conventional medicines, whereas the majority, 334 (79.0%, 95% CI: 74.8–82.7), exhibited a negative attitude. Age, religion, and marital status were significantly associated with attitude. Respondents older than 60 years were significantly more likely to have a positive attitude compared with younger respondents (OR = 3.96, 95% CI: 1.90–8.20, *P* < 0.001). Catholic respondents were less likely to have a positive attitude compared with Protestant respondents. Additionally, single and widowed respondents were more likely to exhibit positive attitudes than married respondents (Table 4).

### Factors associated with practices

Good practices toward conventional medicines were reported by 226 respondents (53.4%, 95% CI: 48.5–58.3), while 197 (46.6%, 95% CI: 41.7–51.5) demonstrated poor practices. Marital status, sex, and religion were significantly associated with practice levels. Single respondents were nearly twice as likely to demonstrate good practices compared with married respondents (OR = 1.97, 95% CI: 1.20–3.30, *P* = 0.012). Female respondents were also more likely to exhibit good practices compared with male respondents (OR = 1.57, 95% CI: 1.00–2.40, *P* = 0.032). Similarly, Catholic respondents showed higher odds of good practices compared with Protestant respondents (OR = 2.16, 95% CI: 1.20–4.00, *P* = 0.015) (Table 4).

### Reasons for preference for traditional treatments

**Fig. 3:**
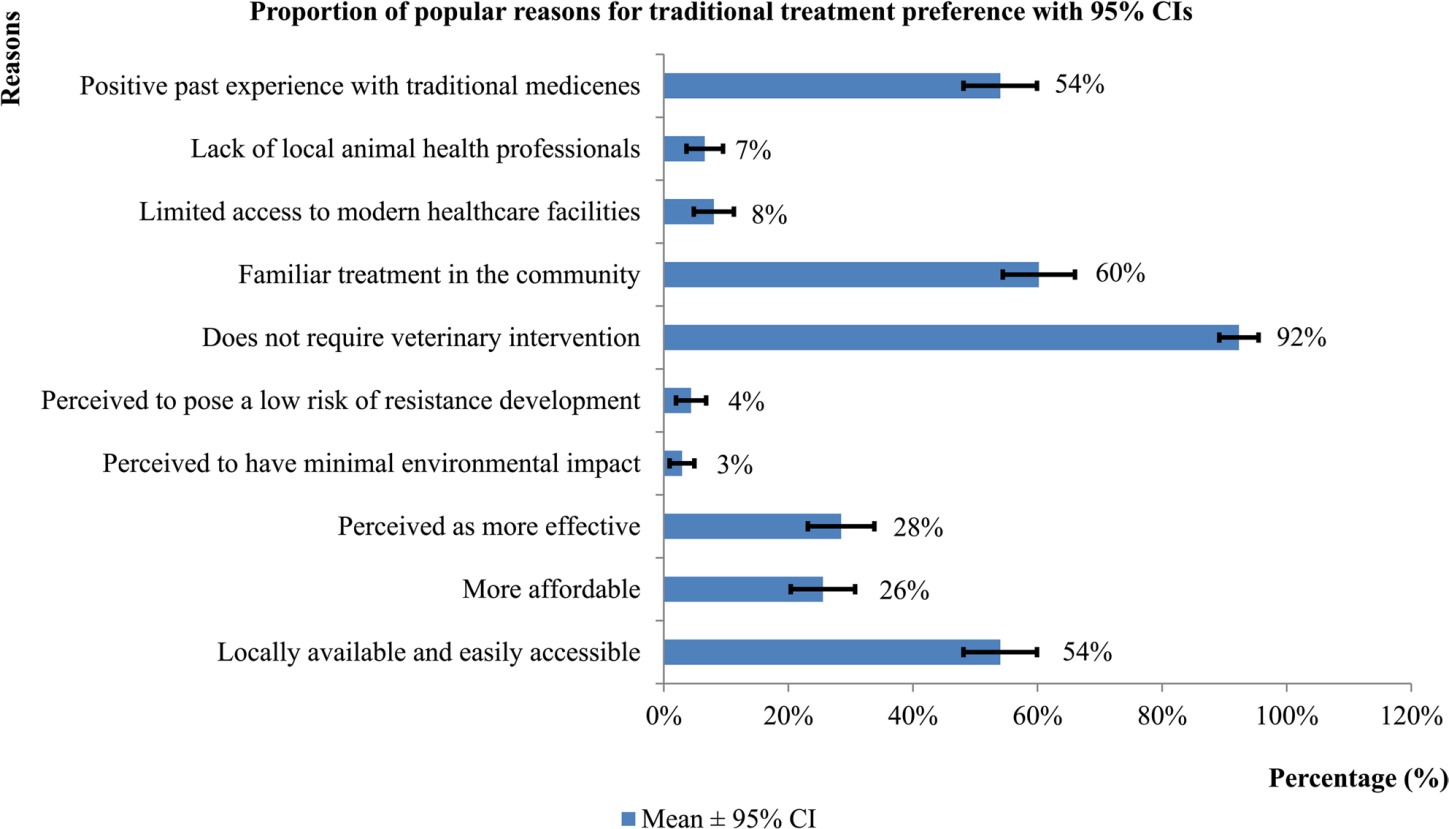
Reasons for the preference for traditional ectoparasite treatments among respondents in Hawassa City, Ethiopia (multiple responses allowed) (*n* = 274)

Out of the total 423 respondents included in the survey, 274 (64.8%, 95% CI: 60.0–69.3) reported using traditional treatments for ectoparasite control. Among these respondents, multiple reasons for preferring traditional treatments were identified. The most frequently cited reason was that traditional treatments did not require veterinary intervention, whereas the perception that they have minimal environmental impact was the least commonly reported reason (Fig. 3).

### Traditional and conventional treatments known in Hawassa City

Out of the total 423 respondents, 274 (64.8%, 95% CI: 60.0–69.3) reported using traditional treatments for ectoparasite control, while 215 (50.8%, 95% CI: 46.0–55.7) reported using conventional treatments. Since multiple responses were allowed, the percentages do not sum to 100%. These findings indicate that traditional and conventional ectoparasite control methods are not mutually exclusive and are often used concurrently.

Among respondents who reported using traditional treatments (*n* = 274), a total of 14 treatment types were identified, including both plant-based and non-plant-based remedies (Table 5). Plant-based treatments were the most frequently reported (73.4%), followed by kerosene (20.8%) and kitchen oil (7.7%). These remedies were mainly applied topically by rubbing onto the skin and hair or administered orally. Other reported treatments included enset products, cow urine, cow dung, fire, wood ash, used engine oil, butter, salt, soap, and petroleum jelly, as well as practices such as burning ticks and smoke-based applications.

**Table 5:** Traditional treatment types reported by ruminant farmers in Hawassa City (multiple responses allowed, *n* = 274))

Treatment	Preparation and Application	Target parasite	Frequency	Percent (%)
Plants	Crushing, squeezing, powdering & drench orally or rub onto hair & skin	Tick, lice, mite, flea	201	73.4
Enset juice	Extracted white sap (juice) from enset pulp rubbed onto affected hair & skin	Lice	8	2.9
Enset fiber	Burned to attack lice with the smoke	Lice	5	1.8
Kerosene	Mixed with water or as it is rubbed onto the affected hair & skin	Tick, lice, flea	57	20.8
Kitchen oil	Rubbed onto the affected hair and skin	Tick, flea	21	7.7
Used engine oil	Rubbed onto the affected hair and skin	Tick	3	1.1
Vaseline	Rubbed onto the affected hair and skin	Lice	2	0.7
Wood ash	Mixed with water and rubbed onto the affected hair & skin	Tick	12	4.4
Cow urine	Wash the affected part	Tick	11	4.0
Cow dung	Mixed with water and rubbed onto the affected hair & skin	Tick	13	4.7
Butter dish	Already used butter dishes burned to attack lice with the smoke	Lice	4	1.5
Salt	Mixed with water and wash the affected hair and skin	Tick, flea	9	3.3
Soap	Mixed with warm water and wash the affected part	Lice, mites	14	5.1
Fire	Burn manually removed ticks	Tick	10	3.6

Percentages are calculated based on the number of respondents reporting traditional treatments (*n* = 274). Multiple responses were allowed; therefore, percentages do not sum to 100%

**Fig. 4:**
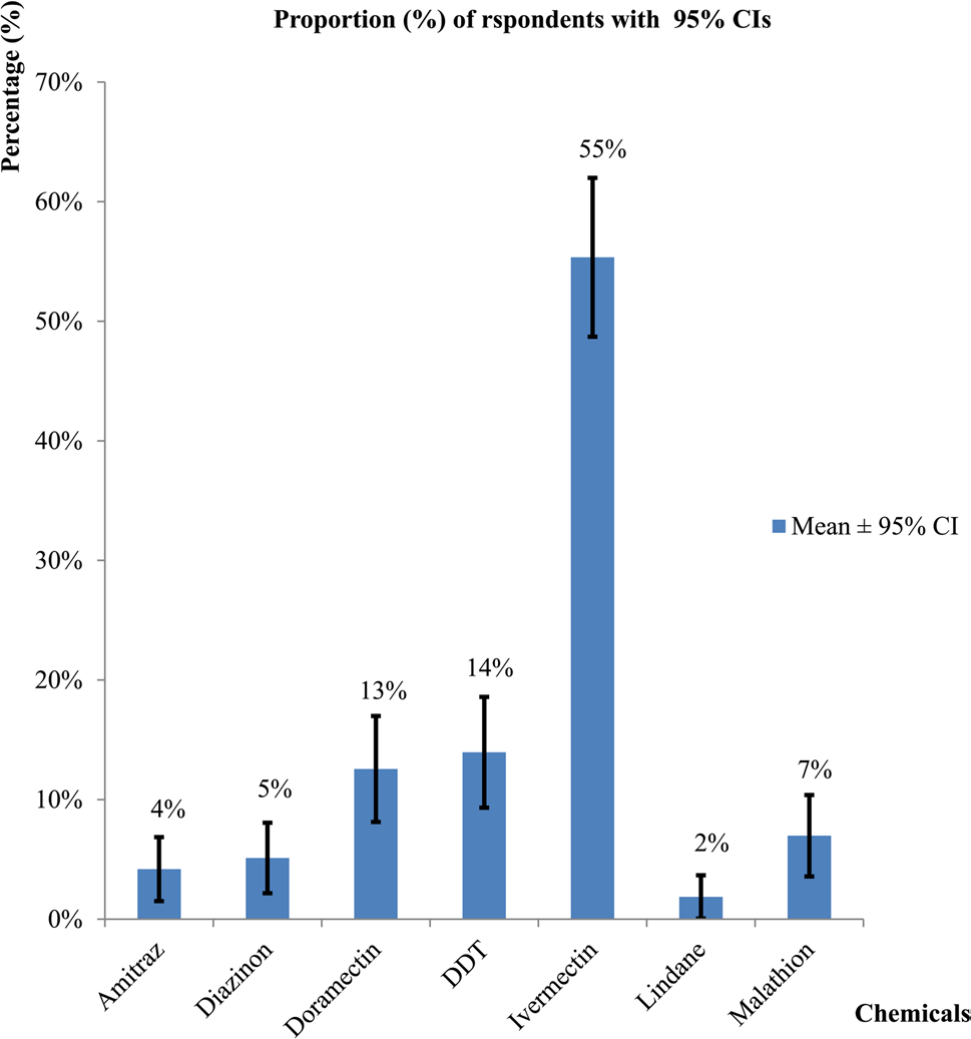
Conventional ectoparasiticides known by respondents in Hawassa City, Ethiopia (multiple responses allowed; *n* = 215)

Among respondents who reported using conventional treatments (*n* = 215), seven acaricides and insecticides were identified: ivermectin, doramectin, diazinon, amitraz, lindane, DDT, and malathion (Fig. 4). Except for DDT and malathion, all were reportedly available in Hawassa during the study period. These findings highlight the coexistence of traditional and conventional ectoparasite control methods and underscore the need for awareness creation, training, and guidance to ensure their safe and effective use.

## Discussion

This study demonstrates that both traditional and conventional ectoparasite control methods are practiced among livestock owners in Hawassa City; however, important gaps in knowledge, attitudes, and practices remain, potentially compromising livestock health and productivity. A relatively small proportion of respondents were aware of both treatment types, which is lower than reports from Benatsemay District, Ethiopia [21] and South Africa [36], reflecting possible urban–rural differences in knowledge transmission. Conversely, a considerable proportion of respondents relied solely on traditional knowledge, a finding higher than that reported in KwaZulu-Natal and Limpopo, South Africa [36, 37], indicating the persistence of indigenous knowledge even in urban settings. Notably, a substantial proportion of respondents lacked any knowledge of ectoparasite treatments, highlighting important gaps that require targeted awareness interventions [38].

All three KAP components for traditional medicines were below optimal levels, consistent with studies reporting the decline of indigenous knowledge due to urbanization and reduced intergenerational transfer [39, 40]. Older, more experienced, and less formally educated farmers demonstrated stronger traditional knowledge, confirming that demographic factors influence knowledge retention [36–40]. In contrast, knowledge of conventional medicines appeared relatively higher, likely reflecting better access to veterinary services in urban settings [22, 41]. However, favorable attitudes toward these products remained limited, suggesting possible concerns or misconceptions regarding their safety or effectiveness. Reported practices were comparatively better than attitudes, indicating that accessibility and availability may encourage use even when perceptions are not fully positive. This pattern highlights that awareness alone does not necessarily translate into appropriate or informed use [22].

Preference for traditional remedies was primarily due to self-administration without veterinary intervention, contrasting with other studies where efficacy or avoidance of chemical residues were key motivators [37, 38, 42]. Participants reported diverse traditional remedies, including plant extracts, enset products, butter, cow urine, kitchen oil, used engine oil, and practices such as burning ticks and smoke application, reflecting a rich ethnoveterinary knowledge base but also potential risks associated with unstandardized practices [42–51]. While some plant-based remedies are effective, inexpensive, and locally accessible, others may pose risks to animals, users, and the environment [43, 46, 47].

Among conventional medicines, ivermectin was the most commonly known, likely due to its broad spectrum, affordability, and availability [22]. Regional preferences varied, with diazinon and amitraz reported in other studies [21, 52], highlighting the need for evidence-based guidance for appropriate and responsible use. Improper practices, such as underdosing, overdosing, repeated use of the same product, and failure to observe withdrawal periods, remain concerns that could reduce efficacy and accelerate resistance [26, 31].

Demographic patterns indicate that traditional knowledge is concentrated among older, more experienced farmers, often engaged in semi-intensive systems, consistent with prior studies [36–40]. In contrast, conventional KAP was influenced by age, gender, marital status, and religion, partially aligning with previous findings [22, 39, 53]. These observations suggest that extension programs should be tailored to specific socio-demographic groups to improve uptake and promote appropriate practices.

Overall, livestock owners tend to combine multiple treatment options depending on availability, experience, and perceived effectiveness. Conventional acaricides are generally more reliable when used correctly, whereas validated traditional remedies may serve as complementary alternatives, particularly in areas with limited access to veterinary services. This underscores the need for integrated awareness programs to promote standardized preparation, appropriate dosage, demonstrated efficacy, and safer use of traditional remedies, alongside the responsible application of conventional medicines.

### Policy and SDG implications

These findings have important implications for Ethiopian livestock policy and broader development priorities. Improving KAP in ectoparasite control contributes to SDG 2 (Zero Hunger) through enhanced livestock productivity and food security [1, 26, 32, 34], as well as SDG 3 (Good Health and Well-being) and SDG 15 (Life on Land) by reducing zoonotic risks and promoting sustainable livestock management practices. Strengthening urban extension services can facilitate the documentation, preservation, and safe integration of traditional knowledge with modern veterinary practices, thereby aligning local interventions with national livestock strategies and global sustainability targets [1, 26, 34].

## Conclusion and recommendations

This study showed that awareness of ectoparasite control methods among ruminant owners in Hawassa City was incomplete. Although many respondents recognized either traditional or conventional methods, fewer were familiar with both approaches, indicating fragmented knowledge that may influence treatment decisions.

A wide range of traditional remedies was reported, including plant-based preparations and non-plant materials such as enset products, kerosene, kitchen oil, used engine oil, Vaseline, wood ash, cow urine, cow dung, butter, salt, soap, burning ticks, and smoke-based practices. Respondents also reported the use of several conventional acaricides, particularly ivermectin, doramectin, diazinon, amitraz, lindane, DDT, and malathion. This diversity reflects the presence of multiple ectoparasite control options in the study area.

Traditional remedies were mainly used because they are affordable, locally available, and culturally familiar. However, low KAP scores indicate that many respondents may not fully understand appropriate preparation methods, effectiveness, or potential risks associated with some practices. Although conventional medicines were widely known and used, their limited positive perception suggests concerns related to cost, access, and correct application.

Overall, the findings indicate that ectoparasite control practices are influenced by information gaps rather than a single preferred approach. Conventional acaricides are more likely to provide reliable control when correctly applied, whereas traditional practices remain important in situations where veterinary access is limited. However, the use of potentially harmful materials such as kerosene and used engine oil highlights the need for urgent educational intervention.

Demographic differences in KAP suggest that age, education, occupation, religion, and farming experience influence how livestock owners obtain and apply ectoparasite control knowledge. Therefore, interventions should be tailored to specific community groups rather than applied uniformly. 

### Recommendations

1. Government livestock and veterinary authorities should strengthen community-based education on safe and effective ectoparasite control practices.
2. Veterinarians and animal health extension workers should provide practical training on correct diagnosis, acaricide selection, dosage, application frequency, and withdrawal periods.
3. Extension programs should discourage unsafe traditional practices, particularly the use of kerosene, used engine oil, and other hazardous substances.
4. Indigenous knowledge on plant-based remedies should be documented and preserved through structured community knowledge-sharing initiatives.
5. Promising traditional remedies should be scientifically evaluated through laboratory and field studies to determine their safety, efficacy, and appropriate use.
6. Further studies should be conducted in other regions of Ethiopia to compare KAP patterns across different production systems and cultural contexts.

### Study limitations

Although this study provides useful baseline information, several limitations should be considered when interpreting the findings. First, the study was conducted among ruminant owners in Hawassa City and focused specifically on ectoparasite control practices; therefore, the findings may not be generalizable to other regions of Ethiopia, livestock production systems, or other animal health conditions.

Second, the cross-sectional design provides only a snapshot of respondents’ knowledge, attitudes, and practices at a single point in time and does not allow for causal inference between socio-demographic factors and KAP outcomes.

Third, the data were based on self-reported information and may be subject to recall bias, social desirability bias, or reporting inaccuracies. In addition, some meaning may have been affected during translation from the local language into English.

Fourth, although a wide range of variables was included, some potentially important factors influencing ectoparasite control practices and decision-making may not have been captured.

Finally, the study documented commonly used traditional and conventional treatments but did not evaluate their efficacy, safety, dosage, or frequency of application. Therefore, the reported treatments should not be interpreted as equally effective or recommended without further experimental validation.

Despite these limitations, efforts were made to enhance data quality and reliability, including the use of a relatively large sample size, pre-tested questionnaires, and data collection supported by trained local language facilitators. 

## Supplementary Information

Supplementary Material 1.

Supplementary Material 2.

## Data Availability

The data presented in this study are available from the corresponding author upon reasonable request.

## Abbreviations

CM: Conventional Medicines
CSA: Central Statistical Agency
DDT: Dichlorodiphenyl Trichloroethane
FAO: Food and Agriculture Organization
KAP: Knowledge, Attitude, and Practice
SDG: Sustainable Development Goals
STATA: Statistical Software for Data Science
TM: Traditional Medicines
