# Supplementary material for: Knowledge, attitudes, and practices related to traditional and conventional ectoparasite control in domestic ruminants: a cross-sectional survey conducted in Hawassa City, Ethiopia
Source: BMC Vet Res. 2026 May 23;22:428. doi: 10.1186/s12917-026-05572-y (PMC13374264; doi:10.1186/s12917-026-05572-y)
Supplement: Supplementary file 2 — Supplementary Material 2. [file 12917_2026_5572_MOESM2_ESM.pdf]

**Questionnaire Cover Page**

**Title:** Knowledge, Attitudes, and Practices Regarding Traditional and Conventional Ectoparasite Control in Domestic Ruminants: A Cross-Sectional Study in Hawassa City, Ethiopia

**Dear Participant**,

We are researchers from Hawassa University conducting a scientific study to assess livestock owners' knowledge, attitudes, and practices regarding the use of traditional remedies and conventional veterinary medicines for controlling ectoparasites in domestic ruminants (cattle, sheep, and goats) in Hawassa City, Ethiopia.

Ectoparasites such as ticks, lice, fleas, and mites reduce animal productivity and may transmit serious diseases. Understanding current management practices is essential for improving animal health, minimizing economic losses, and supporting sustainable livestock production systems.

**The objectives of this study are to**:

- Assess knowledge, attitudes, and practices related to traditional and conventional ectoparasite control
- Identify factors influencing treatment choices
- Document locally used traditional remedies and commercially available veterinary drugs

You have been randomly selected to participate in this study. The questionnaire will take approximately 10–15 minutes to complete.

**Participation in this study is entirely voluntary**. You may decline to answer any question or withdraw from the study at any time without any penalty or consequences. All information collected will be kept strictly confidential and used solely for academic and research purposes. No personal identifiers will appear in any report, publication, or presentation resulting from this study.

By completing this questionnaire, you indicate your informed consent to participate.

Your responses will provide valuable information that may contribute to improving veterinary extension services and community-based animal health interventions.

Thank you very much for your time and cooperation.

Sincerely,

**Dr. Kabech Gedeno**

Principal Investigator

Hawassa University

**Section I: Socio-Demographic Characteristics and Ruminant Farming Practices**

**Part A: Socio-Demographic Characteristics**

1. Date of interview: _____________

2. Telephone number (optional): _____________

3. Kebele: _____________

4. Place of residence:

☐ Urban

☐ Rural

5. Sex:

☐ Male

☐ Female

6. Age (in completed years): _____________

7. Marital status:

☐ Married

☐ Single

☐ Divorced

☐ Widowed

8. Educational level:

☐ No formal education

☐ Primary education

☐ Secondary education

☐ College/University (tertiary education)

9. Primary occupation:

☐ Farmer

☐ Government/private employee

☐ Merchant

☐ Other (specify): ____________

10. Religion (optional):

☐ Orthodox

☐ Muslim

☐ Protestant

☐ Catholic

☐ Other (specify): ____________

Part B: Ruminant Farming Systems and Ectoparasite Management Practices

11. Which ruminant species do you own? (Select all that apply)

☐ Cattle

☐ Sheep

☐ Goats

12. Total number of ruminant animals currently owned (herd size): ____________

13. Farming system:

☐ Intensive

☐ Semi-intensive

☐ Extensive

14. Years of experience in ruminant farming: ____________ years

15. Do you have a separate shelter for your animals?

☐ Yes

☐ No

16. Do you practice regular cleaning of animal shelters?

☐ Yes

☐ No

17. Do you consider ectoparasites a significant problem in your herd?

☐ Yes

☐ No

18. Which ectoparasites are commonly observed in your animals? (Select all that apply)

☐ Ticks

☐ Mites

☐ Lice

☐ Fleas

19. What clinical signs have you observed in animals infested with ectoparasites? (Select all that apply)

☐ Hair loss (alopecia)

☐ Restlessness

☐ Frequent scratching

☐ Weight loss

☐ Skin lesions

20. During which season is ectoparasite infestation most common?

☐ Wet season

☐ Dry season

☐ Both seasons equally

☐ Unsure

21. In your opinion, how do animals most commonly acquire ectoparasites? (Select all that apply)

☐ From pasture or soil during grazing

☐ Through direct contact with infested animals

☐ From contaminated barns/shelters

☐ During transport or at livestock markets

☐ Other (specify): ____________

Part C: Access to Veterinary Services and Treatment Practices

22. Do you have access to veterinary services when needed?

☐ Yes

☐ No

22.1 If yes, what type of veterinary service is primarily accessible to you?

☐ Government veterinary clinic

☐ Private veterinary clinic

☐ Community animal health worker

☐ Veterinary pharmacy/drug vendor only

☐ Other (specify): ____________

23. How many times per year do you treat your animals for ectoparasites (on average)? ____________ times per year

24. Do you implement preventive measures against ectoparasites?

☐ Yes

☐ No

24.1 If yes, which preventive measures do you use? (Select all that apply)

☐ Conventional veterinary medicines (e.g., acaricides, insecticides)

☐ Traditional/herbal remedies

☐ Combination of conventional and traditional methods

☐ Other (specify): ____________

**Section II: Knowledge, Attitudes, and Practices (KAP) Regarding Traditional Ectoparasite Control in Domestic Ruminants**

**1. General Awareness of Traditional Remedies for Ectoparasite Control**

1.1 Have you ever received prior knowledge or training on traditional ectoparasite control in domestic ruminants?

☐ Yes

☐ No

1.1.1 If yes, where did you acquire this knowledge? (Select all that apply)

a) Family members
b) Community members
c) Traditional healers
d) Agricultural extension officers
e) University/College education
f) Scientific publications
g) Social media/online platforms
h) Other (specify): __________

1.2 Are you aware of different types of traditional remedies used for ectoparasite control?

☐ Yes

☐ No

1.2.1 If yes, which types of traditional remedies have you used? (Select all that apply)

a) Medicinal plants/herbs

b) Mineral-based substances (e.g., ash, soil derivatives)

c) Plant-derived oils or extracts

d) Household substances (e.g., salt solutions)

e) Other (specify): __________

1.2.2 For which ectoparasites have these remedies been used? (Select all that apply)

a) Ticks

b) Mites

c) Lice

d) Fleas

e) Other (specify): __________

1.2.3 Which preparation methods are commonly used? (Select all that apply)

a) Boiling in water

b) Soaking/maceration

c) Grinding/crushing into powder

d) Mixing with oil or fat

e) Other (specify): __________

1.2.4 Which application methods are commonly used? (Select all that apply)

a) Topical rubbing/brushing

b) Washing/spraying

c) Dipping in prepared solution

d) Application to housing/environment

e) Other (specify): ___________

1.2.5 Who primarily applies traditional treatments?

a) Farmer (self)

b) Family member

c) Traditional healer

d) Veterinary professional

e) Other (specify): ___________

**2. Knowledge of Traditional Remedies for Ectoparasite Control**

**Response options** (5-point Likert scale):

1 = Strongly disagree, 2 = Disagree, 3 = Neutral, 4 = Agree, 5 = Strongly agree

| **No.** | **Statement** | **1** | **2** | **3** | **4** | **5** |
|---|---|---|---|---|---|---|
| 1 | I know the traditional remedies (plants, minerals, or other materials) used to control ectoparasites in domestic ruminants. | | | | | |
| 2 | I know the specific plant parts or materials required to prepare these remedies (e.g., leaves, roots, bark). | | | | | |
| 3 | I know the appropriate methods for preparing these remedies (e.g., crushing, boiling, soaking, mixing). | | | | | |
| 4 | I know the correct dosage for different ruminant species and age groups. | | | | | |
| 5 | I know the recommended frequency and timing of application. | | | | | |
| 6 | I know the appropriate route of administration (e.g., topical application, washing, spraying). | | | | | |
| 7 | I know how to appropriately combine different remedies or ingredients to enhance their effectiveness. | | | | | |
| 8 | I know how to assess whether a remedy has effectively controlled | | | | | |

| | | | | | | |
|---|---|---|---|---|---|---|
| | ectoparasites. | | | | | |
| 9 | I am aware of potential adverse effects of these remedies on animals (e.g., toxicity, skin irritation). | | | | | |
| 10 | I know how to minimize risks associated with their use. | | | | | |
| 11 | I understand how environmental factors (e.g., rainfall, temperature, season) influence their effectiveness. | | | | | |
| 12 | I know which remedies are most commonly preferred by local farmers or communities. | | | | | |
| 13 | I know local traditional healers or community experts who provide ectoparasite treatments for domestic ruminants. | | | | | |

### 3. Attitudes toward Traditional Remedies for Ectoparasite Control

**Response options** (5-point Likert scale):

1 = Strongly disagree, 2 = Disagree, 3 = Neutral, 4 = Agree, 5 = Strongly agree

| **No.** | **Statement** | **1** | **2** | **3** | **4** | **5** |
|---|---|---|---|---|---|---|
| 1 | Traditional medicines are effective in controlling ectoparasites in domestic ruminants. | | | | | |
| 2 | Traditional remedies reduce the recurrence of ectoparasite infestations. | | | | | |
| 3 | Traditional remedies should be considered as a first-line treatment option. | | | | | |
| 4 | I prefer traditional treatments over conventional veterinary medicines. | | | | | |
| 5 | Traditional remedies are more accessible than conventional anti-parasitic treatments. | | | | | |
| 6 | Traditional medicines are more affordable than conventional anti-parasitic drugs. | | | | | |
| 7 | Traditional treatments are safe for animals. | | | | | |
| 8 | Traditional medicines are easy to prepare and apply. | | | | | |
| 9 | Traditional medicines have minimal negative environmental impact. | | | | | |

| | | | | | | |
|---|---|---|---|---|---|---|
| 10 | Environmental considerations influence my choice of ectoparasite treatment. | | | | | |
| 11 | Traditional medicines can be effectively combined with conventional treatments. | | | | | |
| 12 | Ectoparasites are less likely to develop resistance to traditional medicines. | | | | | |
| 13 | I use traditional medicines when conventional treatments are unavailable. | | | | | |
| 14 | I would recommend traditional medicines to other livestock owners. | | | | | |

**4. Practices Related to Traditional Remedies for Ectoparasite Control**

**Response options** (5-point Likert scale):

1 = Always, 2 = Often, 3 = Sometimes, 4 = Rarely, 5 = Never

| **No.** | **Statement** | **1** | **2** | **3** | **4** | **5** |
|---|---|---|---|---|---|---|
| 1 | I personally prepare traditional medicines before treating my animals. | | | | | |
| 2 | I follow consistent preparation procedures. | | | | | |
| 3 | I determine and apply appropriate dosages. | | | | | |
| 4 | I maintain hygienic conditions during preparation and application. | | | | | |
| 5 | I monitor animals after treatment. | | | | | |
| 6 | I repeat treatments at appropriate intervals when necessary. | | | | | |
| 7 | I treat both animals and their housing environment to prevent reinfestation. | | | | | |

**5. Reasons for Using Traditional Remedies for Ectoparasite Control (Select all that apply)**

| **No.** | **Reasons** | **Mark (✓)** |
|---|---|---|
| 1 | Locally available and easily accessible | |
| 2 | More affordable | |

| | | |
|---|---|---|
| 3 | Perceived as more effective | |
| 4 | Perceived to have minimal environmental impact | |
| 5 | Perceived to pose a low risk of resistance development | |
| 6 | Does not require veterinary intervention | |
| 7 | Familiar treatment in the community | |
| 8 | Limited access to modern healthcare facilities | |
| 9 | Lack of local animal health professionals | |
| 10 | Positive past experience with traditional medicines | |
| 11 | To preserve traditional knowledge | |

**Section III: Knowledge, Attitudes, and Practices Regarding Conventional Ectoparasite Control in Domestic Ruminants**

**1. General Awareness of Conventional Ectoparasite Control Medicines**

1.1 Have you ever received prior knowledge or training on conventional ectoparasite control in domestic ruminants?

☐ Yes

☐ No

1.1.1 If yes, where did you acquire this knowledge? (Select all that apply)

a) Family

b) Community members
c) Agricultural extension officers
d) University/College education
e) Veterinary professionals
f) Scientific publications
g) Social media/online platforms
h) Other (specify): ___________

1.1.2 Are you aware of different types of conventional ectoparasite treatments?

☐ Yes

☐ No

1.1.2.1 Which types of conventional treatments have you used? (Select all that apply)

a) Organophosphates (e.g., diazinon)
b) Synthetic pyrethroids (e.g., flumethrin)
c) Macrocyclic lactones (e.g., ivermectin, doramectin)
d) Amitraz-based products
e) Carbamates or formamidines
f) Insect growth regulators or repellents
g) Other (specify): __________

1.1.2.2 For which ectoparasites are these treatments used? (Select all that apply)

a) Ticks
b) Mites
c) Lice
d) Fleas
e) Other (specify): __________

1.1.2.3 Which preparation procedures are required before use? (Select all that apply)

a) Dilution with water
b) Mixing according to label instructions
c) No preparation required (ready-to-use)
d) Other (specify): __________

1.1.2.4 Which application methods are commonly used? (Select all that apply)

a) Spraying
b) Dipping
c) Pour-on/spot-on
d) Injection

e) Environmental spraying

f) Other (specify): ___________

1.1.2.5 Who usually administers conventional treatments?

a) Farmer (self)

b) Veterinary professional

c) Trained animal health worker

d) Other (specify): ___________

**2. Knowledge of Conventional Ectoparasite Control Medicines**

**Response options** (5-point Likert scale):

1 = Strongly disagree, 2 = Disagree, 3 = Neutral, 4 = Agree, 5 = Strongly agree

| **No.** | **Statement** | **1** | **2** | **3** | **4** | **5** |
|---|---|---|---|---|---|---|
| 1 | I know the main categories of conventional medicines used for ectoparasite control. | | | | | |
| 2 | I know where to obtain approved conventional ectoparasite control medicines. | | | | | |
| 3 | I know qualified professionals who provide conventional ectoparasite treatments. | | | | | |
| 4 | I know the correct dosage, dilution, and treatment intervals for commonly used products. | | | | | |
| 5 | I know the appropriate method of application for each product type. | | | | | |
| 6 | I am aware of potential side effects, withdrawal periods, and residue risks associated with conventional treatments. | | | | | |
| 7 | I understand the environmental risks related to chemical ectoparasite control. | | | | | |
| 8 | I understand the economic implications of using conventional treatments. | | | | | |

**3. Attitudes toward Conventional Ectoparasite Treatments**

**Response options** (5-point Likert scale):

1 = Strongly disagree, 2 = Disagree, 3 = Neutral, 4 = Agree, 5 = Strongly agree

| **No.** | **Statement** | **1** | **2** | **3** | **4** | **5** |
|---|---|---|---|---|---|---|
| 1 | Conventional medicines are effective in controlling ectoparasite infestations. | | | | | |
| 2 | Conventional treatments are safe when properly used. | | | | | |
| 3 | Conventional medicines act quickly to control ectoparasite infestations. | | | | | |
| 4 | Conventional treatments are scientifically trustworthy. | | | | | |
| 5 | Conventional medicines are necessary in cases of severe infestation. | | | | | |
| 6 | The benefits of conventional treatments outweigh their risks. | | | | | |
| 7 | I would recommend conventional medicines to other livestock owners. | | | | | |

**4. Practices Related to Conventional Ectoparasite Treatments**

**Response options** (5-point Likert scale):

1 = Always, 2 = Often, 3 = Sometimes, 4 = Rarely, 5 = Never

| No. | Statement | 1 | 2 | 3 | 4 | 5 |
|---|---|---|---|---|---|---|
| 1 | I use conventional medicines according to veterinary or label instructions. | | | | | |
| 2 | I measure the correct dosage before administration. | | | | | |
| 3 | I apply conventional medicines using recommended methods. | | | | | |
| 4 | I follow safety and hygiene precautions during handling and application. | | | | | |
| 5 | I treat animal housing or the environment when necessary. | | | | | |
| 6 | I monitor animals after treatment to assess effectiveness. | | | | | |

Thank you for your valuable participation in this study.

If you have any additional comments, suggestions, or questions, please provide them below:

______________________________________________________________
